# Super Responders in Plaque Psoriasis: A Real-World, Multi-Agent Analysis Showing Bimekizumab Associated with the Highest Odds of PASI = 0 at Week 12

**DOI:** 10.3390/jcm14207293

**Published:** 2025-10-16

**Authors:** Dominika Ziolkowska-Banasik, Kamila Zawadzinska-Halat, Paulina Basta, Maciej Pastuszczak

**Affiliations:** Clinical Department of Dermatology, Medical University of Silesia, 41-800 Zabrze, Poland; dziolkowska@op.pl (D.Z.-B.); zawadzinskakamila@gmail.com (K.Z.-H.); paulinabasta888@gmail.com (P.B.)

**Keywords:** psoriasis, biologics, bimekizumab, super responders, IL-17 inhibitors, IL-23 inhibitors, real-world evidence, PASI, logistic regression

## Abstract

**Introduction**: Super responders (SRs)—patients achieving complete skin clearance (PASI = 0) soon after biologic initiation—represent a clinically relevant but underexplored phenotype. This study is one of the first real-world, multi-agent analyses comparing SR likelihood across biologic classes in plaque psoriasis. We assessed whether biologic choice predicts SR in routine clinical practice. **Methods**: We performed a retrospective, single-center study of 116 adults with moderate-to-severe plaque psoriasis initiating their first biologic (adalimumab, tildrakizumab, guselkumab, risankizumab, bimekizumab, or secukinumab). SR was defined as PASI = 0 at week 12. SR proportions (exact 95% CIs) were compared using Fisher’s exact tests and odds ratios (ORs). Multivariable logistic regression estimated adjusted associations between biologic and SR, controlling for age, sex, disease duration, BMI, baseline PASI, and prior cyclosporine/acitretin. Sensitivity analyses included Firth bias-reduced regression, the exclusion of sparse drug strata, and an alternative endpoint (PASI ≤ 1 at week 12). **Results**: Overall, 26/116 patients (22.4%) achieved SR. SR proportions differed by agent, highest with bimekizumab (11/17; 64.7%); Fisher’s *p* < 0.001 vs. others; OR = 12.83 (95% CI 4.17–39.50). In adjusted models, bimekizumab remained independently associated with SR (adjusted OR = 17.30; 95% CI 4.62–64.82; *p* = 2.35 × 10^−5^), while other covariates were not significant. **Conclusions**: In this real-world cohort, biologic selection—particularly bimekizumab—was the main determinant of early complete clearance. These findings highlight mechanistic class as a key driver of rapid, deep responses and support prospective validation with harmonized SR definitions and extended follow-up.

## 1. Introduction

Psoriasis is a chronic, immune-mediated inflammatory skin disorder affecting approximately 2–4.4% of the global population [1]. Although primarily manifesting with cutaneous lesions, psoriasis is now recognized as a systemic disease with potential multisystem involvement. The underlying inflammatory burden contributes to an increased risk of comorbidities, particularly cardiovascular and metabolic diseases, underscoring the importance of achieving optimal disease control [2].

The advent of biologic therapies has revolutionized the management of moderate-to-severe plaque psoriasis. By selectively targeting key cytokines such as tumor necrosis factor alpha (TNF-α), interleukin (IL)-17, and IL-23, these agents provide high efficacy and durable skin clearance [3,4]. Nevertheless, despite their proven effectiveness, biologics remain costly and represent a substantial economic burden for healthcare systems worldwide [5,6].

In routine practice, the choice of a specific biologic—whether an anti-TNF, anti-IL-17, or anti-IL-23 agent—is often driven by pragmatic or reimbursement considerations rather than individualized treatment algorithms. In the absence of validated predictive biomarkers, therapeutic decisions are largely empirical, both at initiation and upon treatment failure. Two major challenges persist: primary non-response and secondary loss of efficacy over time. Both scenarios contribute to increased healthcare utilization and reduced quality of life.

Within this context, growing attention has been directed toward a distinct subgroup of patients referred to as “super responders”. This term typically describes individuals who achieve complete skin clearance (PASI = 0) within a short period—often 12–16 weeks—after biologic initiation [7,8]. Emerging evidence suggests that these patients may represent a unique disease endotype characterized by durable responses, prolonged drug survival, and a reduced need for switching, potentially allowing treatment de-escalation [9,10]. Reported prevalence rates of super response generally range between 20 and 30%, depending on study design, definition, population, and the biologic agent investigated [11].

Several studies have attempted to identify clinical predictors of super response—such as younger age, lower body mass index (BMI), shorter disease duration, and bio-naïve status—but most have focused on IL-23 inhibitors, particularly guselkumab [7,12,13]. Consequently, comparative data assessing the likelihood of super response across different biologic classes remain scarce.

To address this knowledge gap, we conducted a real-world, multi-agent analysis aimed at identifying clinical and therapeutic predictors of super response in patients with plaque psoriasis. Specifically, we examined whether the choice of biologic agent influences the probability of achieving early and complete skin clearance and evaluated the consistency of this association across key clinical subgroups.

## 2. Study Design and Patient Population

This was a retrospective, single-center observational study conducted at the Department of Dermatology, Medical University of Silesia. Adult patients with a confirmed diagnosis of moderate-to-severe plaque psoriasis who initiated their first biologic therapy between May 2024 and May 2025 were included. In total, 116 patients met the eligibility criteria and were enrolled.

Inclusion criteria were as follows: age ≥ 18 years; diagnosis of chronic plaque psoriasis for at least 6 months prior to biologic initiation; eligibility for systemic biologic therapy according to Polish national reimbursement regulations, defined as a Psoriasis Area and Severity Index (PASI) score ≥ 10 and a documented lack of efficacy, contraindication, or intolerance to at least two prior conventional systemic agents (cyclosporine, acitretin, or methotrexate); and complete clinical data, including baseline and week-12 PASI scores.

Patients were excluded if they presented with pustular psoriasis, isolated nail psoriasis, or psoriatic arthritis; had active or chronic infections (including HIV, hepatitis B or C, or tuberculosis) or a positive QuantiFERON-TB Gold test; or had coexisting autoimmune or inflammatory diseases (e.g., inflammatory bowel disease, rheumatoid arthritis, systemic lupus erythematosus, or spondyloarthropathies). Additional exclusion criteria included a history of malignancy (except adequately treated basal cell carcinoma), current or recent (within 6 months) participation in interventional clinical trials, or incomplete data regarding treatment response.

Patients were assigned to a specific biologic (adalimumab, tildrakizumab, guselkumab, risankizumab, bimekizumab, or secukinumab) according to drug availability and physician discretion. No randomization or predefined selection algorithm was applied. The study protocol was approved by the Bioethics Committee of the Medical University of Silesia in Katowice (approval number: BNW/NWN/0052/KB1/31/II/24). Written informed consent was obtained from all participants.

## 3. Treatment Protocol

All biologics were administered in accordance with their approved Summary of Product Characteristics (SmPC). Standard dosing regimens were used for all patients regardless of body weight. No dose escalation or increased dosing frequency occurred during the study period.

### 3.1. Data Collection and Clinical Assessment

Baseline demographic and clinical data were extracted from medical records and included age, sex, body mass index (BMI), disease duration (in years), and prior exposure to systemic therapies (methotrexate, cyclosporine, or acitretin). Notably, methotrexate exposure was universal (100%), while variability was observed only for cyclosporine and acitretin.

Psoriasis severity was assessed using the PASI score at baseline and week 12 after biologic initiation. Assessments were independently performed by two board-certified dermatologists experienced in psoriasis management. The final PASI values used for analysis were calculated as the arithmetic mean of the two independent assessments.

### 3.2. Definition of Super Response

Super response (SR) was defined as complete skin clearance (PASI = 0) at week 12 following biologic initiation.

## 4. Statistical Analysis

Continuous variables were summarized as medians (minimum–maximum) and compared using the Mann–Whitney U test. Categorical variables were summarized as counts and percentages and compared using the chi-squared or Fisher’s exact test, as appropriate.

For drug-level comparisons of SR (PASI = 0 at week 12), SR proportions for each biologic agent were reported with exact Clopper–Pearson 95% confidence intervals (CIs). Differences versus all other agents combined were tested using two-sided Fisher’s exact tests. Exact conditional odds ratios (ORs) with 95% CIs were derived from 2 × 2 tables. In the presence of zero cells (e.g., no SRs for a given agent), exact confidence limits were reported and the point estimate was interpreted cautiously; a 0.5 Haldane–Anscombe continuity correction was applied only for forest-plot visualization.

Multivariable logistic regression was performed to assess independent associations between biologic agent and SR, adjusting for age, sex, BMI, disease duration, baseline PASI, and prior exposure to cyclosporine or acitretin. Methotrexate was not entered as a covariate due to the lack of variability (100% prior exposure). Adjusted ORs with 95% CIs were reported, and statistical significance was set at two-sided *p* < 0.05.

Analyses were performed in Python v3.11 using the pandas, SciPy, and statsmodels packages. Firth bias-reduced logistic regression was implemented with the statsmodels and lifelines libraries.

Sensitivity analyses were prespecified to assess robustness with respect to sparse data and endpoint definition:

(i) bias-reduced logistic regression using Firth’s penalized likelihood and identical covariates as the main model; (ii) repetition of drug-level comparisons excluding agents with ≤2 SRs to reduce separation bias; and (iii) redefinition of the endpoint as PASI ≤ 1 at week 12, followed by identical univariable and multivariable analyses.

To further validate the “versus all others” comparison, two complementary sensitivity analyses were performed. First, pairwise Fisher’s exact tests were calculated between bimekizumab and each comparator (adalimumab, guselkumab, risankizumab, secukinumab, and tildrakizumab), with exact ORs, 95% CIs, and two-sided *p*-values reported (Supplementary Table S1). Second, a multivariable logistic regression model was re-estimated using secukinumab—the largest treatment group—as the reference, adjusting for the same covariates (Supplementary Table S2). Both analyses yielded concordant inferences, confirming that the pooled “versus all others” approach did not bias results.

Exploratory treatment-by-covariate interaction models (bimekizumab × age, BMI, disease duration, baseline PASI, sex, or prior treatment) were fitted separately, with other covariates fixed at cohort medians (continuous) or modes (categorical). There were no missing data for baseline or week-12 PASI scores; all enrolled patients had complete records and were included in the final analysis. No multiplicity adjustments were applied.

## 5. Results

### 5.1. Overall Cohort

A total of 116 patients with moderate-to-severe plaque psoriasis treated with biologics were included in the final analysis. Among them, 26 patients (22.4%) achieved a super response (SR), defined as PASI = 0 at week 12 of treatment.

### 5.2. Baseline Characteristics

Prior methotrexate exposure was universal (100%), precluding its inclusion as a covariate in multivariable models. Baseline demographic and clinical characteristics did not differ significantly between super responders and non-super responders (Table 1). Median age was 45 years (range 21–73) among super responders and 48 years (range 18–79) among non-super responders (*p* = 0.3). No significant differences were observed for sex distribution, disease duration, BMI, baseline PASI, or prior systemic treatments (methotrexate, cyclosporine, acitretin).

### 5.3. Association Between Biologic Treatment and Super Response

Super response proportions at week 12 varied across biologic agents (Table 2). The highest SR proportion was observed with bimekizumab (11/17; 64.7%). Fisher’s exact test confirmed a significantly higher SR rate with bimekizumab versus all other agents (*p* < 0.001), corresponding to an exact odds ratio (OR) = 12.83 (95% CI 4.17–39.50).

In contrast, no SRs were recorded with adalimumab (0/16); Fisher’s exact test indicated a lower SR rate (*p* = 0.0428), although the 95% CI for the OR included 1.0, reflecting uncertainty from the zero-event cell. For the remaining agents, exact ORs had wide CIs overlapping 1.0 and non-significant Fisher’s *p*-values (guselkumab *p* = 0.331; risankizumab *p* = 1.000; secukinumab *p* = 0.087; tildrakizumab *p* = 0.369).

To confirm that pooling all non-bimekizumab biologics as a single comparator did not bias the main findings, additional sensitivity analyses were performed. Pairwise Fisher’s exact tests comparing bimekizumab with each individual agent confirmed significantly higher SR proportions for bimekizumab across all comparisons (Supplementary Table S1). Similarly, in a multivariable logistic regression model using secukinumab as the reference drug (Table 3, Supplementary Table S2), bimekizumab remained independently associated with a markedly greater likelihood of achieving SR after adjustment for age, sex, BMI, disease duration, baseline PASI, and prior systemic therapies. The primary pooled-comparator analysis yielded concordant results.

### 5.4. Multivariable and Sensitivity Analyses

In adjusted multivariable logistic regression (including age, sex, BMI, disease duration, baseline PASI, and prior cyclosporine or acitretin), treatment with bimekizumab was independently associated with higher odds of SR (adjusted OR = 17.30; 95% CI 4.62–64.82; *p* = 2.35 × 10^−5^), whereas none of the clinical covariates showed significant associations.

**Figure 1:**
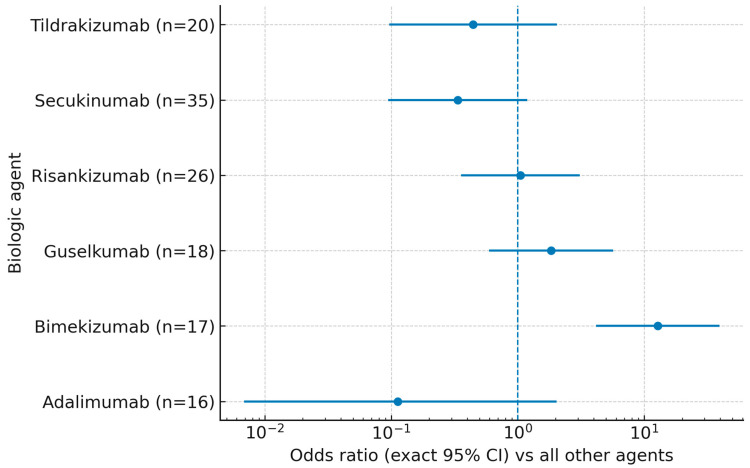
Super response (PASI = 0 at week 12) by biologic agent: exact odds ratios (95% CIs) versus all other agents. Points denote exact ORs from 2 × 2 tables; error bars show exact 95% CIs; the vertical dashed line marks OR = 1. For zero-event strata, a 0.5 continuity correction was applied only for plotting. *n* indicates the number of patients treated with each agent.

As visualized in Figure 1, the forest plot of exact odds ratios (95% CIs) mirrors these findings: only bimekizumab displayed a CI entirely to the right of unity, whereas those for guselkumab, risankizumab, secukinumab, and tildrakizumab crossed 1.0. The adalimumab point estimate, displayed with a 0.5 continuity correction for plotting purposes (zero events), had an exact CI spanning 1.0. Sample sizes for each agent are shown along the *y*-axis.

Sensitivity analyses yielded consistent inferences. In the Firth bias-reduced model, bimekizumab remained independently associated with SR; excluding treatment groups with very few SRs did not alter conclusions. When the endpoint was redefined as PASI ≤ 1 at week 12, overall response proportions increased but the between-agent ranking remained unchanged.

### 5.5. Exploration of Clinical Predictors by Treatment Group

Stratified analyses revealed significant heterogeneity in age among super responders across treatment groups. Those treated with risankizumab had the highest median age (62 years), whereas SRs receiving tildrakizumab or bimekizumab were youngest (29 and 35 years, respectively; *p* = 0.0082).

### 5.6. Interaction Analysis Between Bimekizumab and Clinical Variables

To examine whether the association between bimekizumab and SR was modified by specific clinical factors, separate interaction models were constructed for each variable. None of the interaction terms reached statistical significance, indicating that the effect of bimekizumab was not significantly modified by age (*p* = 0.368), sex (*p* = 0.606), BMI (*p* = 0.565), disease duration (*p* = 0.749), baseline PASI (*p* = 0.542), or prior systemic therapy.

**Figure 2:**
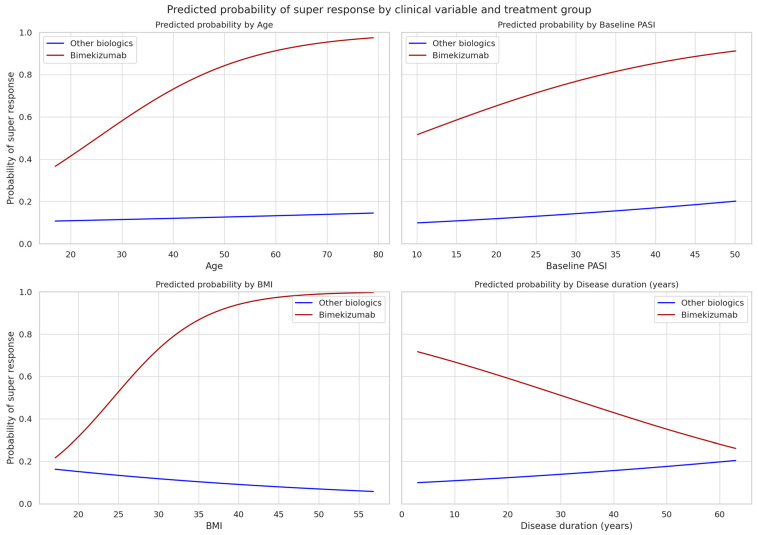
Model-adjusted predicted probability of super response (PASI = 0 at week 12) by age, baseline PASI, BMI, and disease duration for patients treated with bimekizumab (red) versus all other biologics (blue). Curves are derived from separate logistic regression models including treatment, the respective clinical variable, and their interaction, with the remaining covariates held at cohort medians/modes. Interaction terms were not statistically significant; plots are exploratory. Confidence bands are omitted for clarity.

These analyses were prespecified as exploratory; no multiplicity adjustments were applied. Nonetheless, visual inspection of predicted-probability plots (Figure 2) suggested subtle trends toward higher SR probability with increasing age, BMI, and disease duration among bimekizumab-treated patients—patterns not observed with other biologics.

## 6. Discussion

To our knowledge, this real-world study is among the first to compare multiple biologic agents head-to-head with respect to the likelihood of achieving a super response (SR) in plaque psoriasis [7,11,14,15]. Previous reports have typically focused on single agents—most often within the IL-23 class, particularly guselkumab [10,12,13]—or examined predictors of SR across mixed cohorts rather than across mechanistically distinct drugs [7,12,15]. Only a few comparative analyses extend beyond single-drug observations, and these usually omit TNF-α inhibitors, limiting insight into class-level differences [14,16].

There is no universally accepted definition of SR in psoriasis, complicating cross-study comparisons. Some trials defined SR as PASI = 0 at week 20 or 28, others at weeks 12 or 24, or as PASI = 0 at week 20 maintained at week 28; registry frameworks have employed sustained low disease activity over longer follow-up among first-line biologic users [7,9,11]. Despite this heterogeneity, the concept remains clinically meaningful. Patients who clear completely early tend to sustain deep responses, remain longer on therapy, switch less frequently, and—in some cases—can be managed with de-escalated dosing without loss of control [9,10]. Across published cohorts, SR prevalence typically ranges from 20 to 30%, depending on the definition, population, and biologic studied [9,10,11,12,17,18].

Evidence regarding baseline predictors of SR is inconsistent. A relatively consistent finding is that bio-naïve patients are more likely to achieve SR, whereas prior biologic exposure and longer disease duration decrease this probability [9,10,11,12,13]. In contrast, data on BMI are heterogeneous: some studies associate lower BMI with SR [12,17], others show no relationship, and still others report a threshold effect (e.g., reduced response in patients with BMI > 30), possibly due to functional underdosing under fixed-dose regimens in severe obesity [11]. Age-related effects are similarly variable, potentially influenced by comorbidities, polypharmacy, and altered pharmacokinetics with aging [10,12,18]. These inconsistencies emphasize the need for models integrating both drug mechanism and patient phenotype rather than focusing on isolated clinical predictors [7,9].

System-level and cohort characteristics also influence outcomes. In our study, all patients had prior methotrexate exposure before initiating a biologic, which precluded its inclusion as an independent predictor and may have selected for more treatment-refractory disease. Furthermore, all patients were bio-naïve by design, so findings from biologic-experienced populations could not be assessed. National access regulations likely narrowed baseline variability: in Poland, biologics are prescribed for patients with PASI ≥ 10 and documented lack of efficacy, contraindication, or intolerance to at least two systemic agents (usually methotrexate, cyclosporine, or acitretin). Such criteria enrich cohorts for more severe or refractory disease and may attenuate observable effects of clinical covariates.

Within this framework, no independent associations were detected between SR and age, sex, baseline PASI, disease duration, or BMI. Several explanations are possible. First, the drug effect likely dominates: bimekizumab exhibited a strong effect size that could mask smaller influences of clinical covariates. This observation aligns with prior evidence showing that dual IL-17A/IL-17F inhibition achieves faster and deeper skin clearance compared with single-axis IL-17 blockade [3,8,19].

The between-agent pattern observed in this study is biologically plausible. Bimekizumab neutralizes both IL-17A and IL-17F, leading to broader suppression of IL-17–driven inflammation and providing a mechanistic rationale for the higher likelihood of early complete clearance observed in our cohort [3,8,19]. In contrast, IL-23p19 inhibitors (risankizumab, guselkumab, tildrakizumab) act upstream by reducing Th17 maintenance and downstream IL-17/IL-22 production; although they demonstrate excellent long-term efficacy, their early (week-12) trajectory tends to be less steep in both trials and real-world settings. TNF-α inhibition (adalimumab) acts on a broader inflammatory cascade but is generally associated with slower or less frequent complete responses compared with direct IL-17-axis blockade. These pharmacodynamic distinctions are consistent with our finding that dual IL-17A/IL-17F inhibition was the strongest and independent predictor of week-12 SR.

The consistent result across all analyses was that bimekizumab independently predicted SR. This finding is in line with earlier reports showing that dual IL-17A/IL-17F blockade induces rapid and profound clearance, whereas demographic variables such as age or BMI rarely modify this effect [8,12,17,19]. Collectively, these results suggest that drug mechanism—rather than baseline clinical characteristics—is the primary determinant of early complete clearance. This supports the concept that SR may represent a distinct psoriatic endotype that is robust to clinical heterogeneity and can be sustained even with less frequent dosing [9,10].

Our findings regarding bimekizumab’s early efficacy are consistent with results from pivotal phase-III trials and real-world evidence. In the BE RADIANT trial, bimekizumab achieved PASI = 0 in 61.7% of patients at week 16, significantly outperforming secukinumab [19]. Similarly, in BE VIVID, PASI = 0 was reached by 59.3% of participants at week 16 [20]. Real-world studies have reported that 60–70% of patients achieve PASI 90–100 within 8–12 weeks of treatment initiation [8]. Consistent with these data, the 64.7% SR rate observed in our cohort closely mirrors the rapid, deep clearance described across both controlled and observational studies.

Recent real-world research further supports these findings. In a large Italian multicenter cohort (IL PSO), complete clearance (PASI = 0) was achieved by 43.3% of patients at week 4 and 75.4% at week 16, paralleling phase-III outcomes [21]. In a 36-week comparative study, bimekizumab yielded higher PASI = 0 rates than brodalumab at week 4 (41.5% vs. 23.6%) and week 16 (67.9% vs. 48.6%), underscoring its faster onset and deeper early response [22]. Complementing these observations, a focused IL PSO analysis characterized the “super responder” phenotype under bimekizumab, demonstrating a high proportion of early SRs and distinguishing clinical differences between SRs and non-SRs—further reinforcing the biological plausibility of early clearance with dual IL-17 blockade [23]. Collectively, these studies align with our data and highlight that mechanism-of-action choice—particularly dual IL-17A/IL-17F inhibition—is a key driver of early complete clearance in clinical practice.

From a clinical perspective, early achievement of PASI = 0 may serve as a pragmatic marker of optimal drug–patient matching. Recognizing SRs early could enable clinicians to optimize long-term biologic survival, avoid unnecessary treatment switching, and individualize maintenance dosing schedules. This strategy aligns with a broader movement toward precision-guided, time-sensitive treatment goals in chronic inflammatory skin diseases.

Beyond psoriasis, similar trends are emerging across dermatology. In atopic dermatitis, for instance, rapid symptom relief and ease of administration have been linked to improved adherence and patient satisfaction, as shown with once-daily oral agents such as abrocitinib [24]. Although our study focused on injectable biologics, these parallel developments illustrate a unifying dermatologic paradigm: early, complete responses represent both therapeutic potency and patient-centered value. This underscores the need for harmonized SR definitions and comparative studies across diseases to better align efficacy, onset, and treatment burden.

This study has limitations but also notable strengths. Its retrospective, single-center design and modest sample size introduce potential selection bias and residual confounding. Nonetheless, the cohort was clinically homogeneous, consisting entirely of bio-naïve patients initiating biologics under uniform access criteria, which enhanced internal consistency and minimized variability. Cross-country differences in access and reimbursement may limit generalizability and partly explain discrepancies with other cohorts. Prior methotrexate exposure was universal (100%), precluding its use as a covariate. Group sizes were uneven, with zero events for adalimumab leading to quasi-separation and wide confidence intervals for some estimates. Our endpoint at week 12 differed from later harmonized SR timepoints, and 52-week durability data were unavailable. We also lacked therapeutic drug monitoring and immunogenicity assessments, which could clarify pharmacokinetic variability in selected subgroups.

An additional limitation is the potential bias introduced by comparing agents with distinct mechanisms of action. It is well established that IL-17 inhibitors—particularly bimekizumab, which blocks both IL-17A and IL-17F—achieve faster clinical responses than IL-23p19 or TNF-α inhibitors. This pharmacodynamic disparity likely contributed to the between-agent differences observed in week-12 SR rates. Nevertheless, the study’s primary aim was not to rank agents by efficacy but to illustrate how mechanism-of-action selection may influence the likelihood of early complete clearance in real-world settings. Recognizing these class-related patterns provides clinically relevant insight for optimizing early biologic therapy, while underscoring the need for mechanism-matched, head-to-head studies with harmonized endpoints.

Despite these limitations, the study’s strengths include its real-world design, inclusion of multiple biologic classes with standardized clinical assessments, and use of robust statistical methods to mitigate small-sample effects. Together, these features yield novel insights into early, mechanism-specific treatment dynamics in psoriasis and provide a rationale for future multicenter, prospective validation.

Prospective multicenter studies employing harmonized SR definitions and 52-week follow-up are warranted to confirm these results. Future designs should explicitly test drug-by-phenotype interactions and incorporate biomarker data to advance endotype-driven therapy. Additionally, evaluating dose personalization strategies—such as weight-based adjustments or therapeutic drug monitoring—may further enhance SR rates, particularly among patients with high BMI.

## Figures and Tables

**Table 1:** Baseline characteristics of the study cohort by super response status.

	Super Responders (SR); *n* = 26	Non-Super Responders (nSR); *n* = 90	*p*-Value
age; years	45 (21–73)	48 (18–79)	0.36
females; (%)	8 (29.6)	34 (37.3)	0.54
disease duration; years	14 (3–58)	18 (3–63)	0.46
BMI	28.3 (17.5–56.8)	27.4 (17.1–49.6)	0.52
baseline PASI	22.4 (10.9–42)	21.9 (10.1–50.1)	0.91
prior MTX; (%)	26 (100)	90 (100)	
prior CyA; (%)	22 (84.6)	75 (83.1)	0.82
prior acitretin; (%)	11 (42.3)	43 (47.8)	0.73

Data are given as median (min-max) or otherwise stated. Prior MTX = 100% across groups; variable omitted from multivariable models due to no between-group variance. BMI; body mass index, PASI; Psoriasis area and severity index, MTX; methotrexate, CyA; cyclosporine.

**Table 2:** Super response at week 12 by biologic agent: absolute proportions and exact odds ratios (vs all other agents).

Drug	SR/N (%)	SR Rate 95% CI	Exact OR vs. Others (95% CI)	Fisher *p*-Value
adalimumab	0/16 (0.0)	0.0 (0.0–20.6)	0 (0.01–2.03)	0.04 *
bimekizumab	11/17 (64.7)	64.7 (38.3–85.8)	12.83 (4.17–39.50)	0.00001 *
guselkumab	5/18 (27.8)	27.8 (9.7–53.5)	1.84 (0.59–5.68)	0.33
risankizumab	5/26 (19.2)	19.2 (6.6–39.4)	1.05 (0.36–3.09)	1.00
secukinumab	3/35 (8.6)	8.6 (1.8–23.1)	0.34 (0.09–1.19)	0.09
tildrakizumab	2/20 (10.0)	10.0 (1.2–31.7)	0.44 (0.10–2.04)	0.37

Super response (PASI = 0 at week 12) across biologic agents. Shown are absolute proportions with exact Clopper–Pearson 95% CIs and exact odds ratios versus all other agents with two-sided Fisher’s exact *p*-values. For cells with zero events, exact CIs are reported; a 0.5 continuity correction was used only for forest-plot visualization. SR; super response. Significant *p*-values (*p* < 0.05) are marked with an asterisk for clarity.

**Table 3:** Multivariable logistic regression for super response (PASI = 0 at week 12). Values are odds ratios (OR) with 95% confidence intervals (CI).

	Bimekizumab *n* = 17		*p*	Guselkumab *n* = 18		*p*	Risankizumab *n* = 26		*p*	Secukinumab *n* = 35		*p*	Tildrakizumab *n* = 20		*p*
	SR *n* = 11	nSR *n* = 6		SR *n* = 5	nSR *n* = 13		SR *n* = 5	nSR *n* = 21		SR *n* = 3	nSR *n* = 32		SR *n* = 2	nSR *n* = 18	
age; years	35 (21–51)	25.5 18–49)	0.2	46 (27–48)	49 (26–60)	0.3	62 (51–73)	55 (24–72)	0.08	44 (34–53)	42 (24–73)	0.7	29 (24–34)	43 (25–57)	0.1
females; (%)	3 (27.3)	4 (66.7)	0.2	1 (20)	6 (46.2)	0.3	1 (20)	6 (28.6)	0.7	2 (66.7)	14 (43.8)	0.6	1 (50)	4 (22.2)	0.7
disease duration; years	10 (5–22)	10.5 (4–31)	0.9	17 (3–32)	18 (5–27)	0.8	33 (28–58)	25 (5–52)	0.02	27 (4–30)	14 (3–63)	0.9	6 (6–6)	17 (4–37)	0.1
BMI	29.4 (20.1–56.8)	24.5 (19.5–31.5)	0.1	27.7 (22.5–38.4)	27.7 (23.2–42.5)	0.6	31 (23.9–33.7)	27.4 (21.8–38.2)	0.7	27.5 (20.8–32.1)	29.9 (19.6–49.6)	0.6	22.9 (17.5–28.2)	26.8 (18.4–35.6)	0.6
baseline PASI	18 (11.3–38.1)	14.4 (11.3–30.4)	0.4	22.1 (18.9–42)	21 (10.2–34)	0.4	24 (22.4–33.6)	25.2 (11.6–40.5)	0.9	17.7 (13.6–22.5)	23.8 (14.7–50.1)	0.06	12.8 (10.9–14.6)	16 (10.1–27.6)	0.3
prior MTX; (%)	11 (100)	6 (100)		5 (100)	13 (100)		5 (100)	21 (100)		3 (100)	33 (100)	0.3	2 (100)	18 (100)	
prior CyA; (%)	9 (81.8)	4 (66.7)	0.6	4 (80)	9 (69.2)	0.7	5 (100)	19 (90.5)	0.2	1 (33.3)	26 (81.2)	0.3	2 (100)	14 (77.8)	0.04
prior acitretin; (%)	5 (45.5)	3 (50)	0.9	2 (40)	6 (46.2)	0.8	0 (0)	3 (14.3)	0.08	2 (66.7)	14 (43.8)	0.6	2 (100)	8 (44.4)	0.001

Data are given as median (min-max) or otherwise stated. Within-drug *p*-values represent exploratory, descriptive comparisons only. Prior MTX = 100% across groups; variable omitted from multivariable models due to no between-group variance. BMI; body mass index, PASI; Psoriasis area and severity index, MTX; methotrexate, CyA; cyclosporine.

## Data Availability

The original contributions presented in this study are included in the article. Further inquiries can be directed to the corresponding author.

## Supplementary Materials

The following supporting information can be downloaded at: https://www.mdpi.com/article/10.3390/jcm14207293/s1, Table S1: Pairwise comparisons of super response (PASI = 0 at week 12) between bimekizumab and each comparator (Fisher’s exact test); Table S2: Multivariable logistic regression model with secukinumab as reference agent.
