# Supplementary material for: Super Responders in Plaque Psoriasis: A Real-World, Multi-Agent Analysis Showing Bimekizumab Associated with the Highest Odds of PASI = 0 at Week 12
Source: J Clin Med. 2025 Oct 16;14(20):7293. doi: 10.3390/jcm14207293 (PMC12565345; doi:10.3390/jcm14207293)
Supplement: Supplementary file 1 [file jcm-14-07293-s001.zip › jcm-3921707-supplementary.pdf]

Supplementary Table S1. Pairwise comparisons of super response (PASI = 0 at week 12) between bimekizumab and each comparator (Fisher's exact test).

Comparison	SR (%)	Comparator SR (%)	Exact OR (95% CI)	Fisher's p-value
bimekizumab vs adalimumab	64.7 (11/17)	0.0 (0/16)	∞ (4.8–∞)*	8.7×10^{-5}
bimekizumab vs guselkumab	64.7 (11/17)	27.8 (5/18)	4.83 (1.05–22.3)	0.044
bimekizumab vs risankizumab	64.7 (11/17)	19.2 (5/26)	7.86 (2.0–30.7)	0.0040
bimekizumab vs secukinumab	64.7 (11/17)	8.6 (3/35)	20.6 (4.2–100.1)	4.8×10^{-5}
bimekizumab vs tildrakizumab	64.7 (11/17)	10.0 (2/20)	16.3 (2.9–91.8)	0.0013

Values represent exact odds ratios (OR) with corresponding 95% confidence intervals (CI) and two-sided Fisher's exact p-values.

* For adalimumab, an infinite OR arises from a zero-event comparator; exact confidence limits are reported.

SR; super response

Supplementary Table S2. Multivariable logistic regression model with secukinumab as reference agent.

**variable**	**adjusted OR (95% CI)**	**p-value**
**bimekizumab (vs secukinumab)**	18.7 (4.9–70.8)	1.9×10^{-5}
guselkumab (vs secukinumab)	2.0 (0.5–8.1)	0.33
risankizumab (vs secukinumab)	1.1 (0.3–4.0)	0.89
tildrakizumab (vs secukinumab)	0.7 (0.1–3.9)	0.72
adalimumab (vs secukinumab)	0.1 (0.01–1.2)	0.07
age (per year)	0.99 (0.95–1.03)	0.61
female sex	1.2 (0.5–3.0)	0.67
BMI (per kg/m^2)	0.98 (0.92–1.05)	0.53
disease duration (per year)	0.99 (0.96–1.03)	0.68
baseline PASI (per unit)	1.01 (0.95–1.07)	0.73
prior cyclosporine	0.92 (0.36–2.35)	0.86
prior acitretin	0.88 (0.31–2.53)	0.82

The model included all biologic agents and was adjusted for age, sex, BMI, disease duration, baseline PASI, and prior exposure to cyclosporine or acitretin. Odds ratios (OR) with 95% confidence intervals (CI) and two-sided p-values are shown.
